# Research progress of AMP-activated protein kinase and cardiac aging

**DOI:** 10.1515/biol-2022-0710

**Published:** 2023-08-29

**Authors:** Zhengqi Qiu, Yufei Li, Yancheng Fu, Yanru Yang

**Affiliations:** Faculty of Medicine, Macau University of Science and Technology, Taipa, Macao SAR 999078, China; Guangdong Key Laboratory of Genome Stability and Human Disease Prevention, Carson International Cancer Center, Department of Biochemistry and Molecular Biology, School of Basic Medical Sciences, Health Science Center, Shenzhen University, Shenzhen 518060, China

**Keywords:** AMP-activated protein kinase, aging, cardiac senescence, autophagy, reactive oxygen species

## Abstract

The process of aging is marked by a gradual deterioration in the physiological functions and functional reserves of various tissues and organs, leading to an increased susceptibility to diseases and even death. Aging manifests in a tissue- and organ-specific manner, and is characterized by varying rates and direct and indirect interactions among different tissues and organs. Cardiovascular disease (CVD) is the leading cause of death globally, with older adults (aged >70 years) accounting for approximately two-thirds of CVD-related deaths. The prevalence of CVD increases exponentially with an individual’s age. Aging is a critical independent risk factor for the development of CVD. AMP-activated protein kinase (AMPK) activation exerts cardioprotective effects in the heart and restores cellular metabolic functions by modulating gene expression and regulating protein levels through its interaction with multiple target proteins. Additionally, AMPK enhances mitochondrial function and cellular energy status by facilitating the utilization of energy substrates. This review focuses on the role of AMPK in the process of cardiac aging and maintaining normal metabolic levels and redox homeostasis in the heart, particularly in the presence of oxidative stress and the invasion of inflammatory factors.

## 1 Introduction

Cardiovascular disease (CVD) has long been one of the leading causes of death worldwide, as a non-communicable disease causing over 17.3 million deaths per year. Models suggest that the number of deaths could increase to over 23.6 million per year by 2030 [1]. Aging is an independent risk factor for the development of CVD [2]. Cardiac aging is closely associated with time-dependent alterations in cellular metabolism, cardiomyocyte dysfunction (or senescence), and increased occurrence of tissue scarring (fibrosis). Ultimately these events can induce cardiac remodeling stress and potentially initiate heart failure [3–6].

Senescent cardiomyocytes exhibit various characteristic features, including DNA damage, endoplasmic reticulum stress, mitochondrial dysfunction, contractile dysfunction, and the expression of a senescence-associated secretory phenotype (SASP) [7]. Increased cardiac metabolic demand can exacerbate energy production imbalances and oxidative damage [8]. Previous studies have shown that AMP-activated protein kinase (AMPK) regulates mitochondrial biogenesis through the peroxisome proliferator-activated receptor gamma coactivator 1-alpha (PGC1α) signaling pathway. This regulation enhances oxidative mitochondrial metabolism and serves as an important regulator of cardiac metabolism, functioning in both normal and ischemic conditions [9–12]. In addition, AMPK activation leads to substantial inhibition of the mTOR signaling pathway, which effectively reduces apoptosis [13,14]. AMPK also increases autophagy levels via Unc-51 like autophagy activating kinase 1 (ULK1) and reduces tissue fibrosis by inhibiting transforming growth factor beta (TGF-β) signaling [15]. Thus, AMPK activation is thought to play a significant cardioprotective role against cardiotoxicity and is closely associated with the cardiac remodeling process. Furthermore, proteins such as Humanin and SIRT1 are implicated in the cardioprotective effects of AMPK, indicating their participation in AMPK-mediated processes [16–18]. A substantial body of evidence supports the notion that AMPK serves as a metabolic hub, contributing to the improvement of heart health. However, as a heterologous protein complex found in numerous cells and organs, the precise role of AMPK is still being further elucidated [19]. The information compiled in this review will serve as a valuable reference for researchers studying AMPK and is anticipated to contribute to future experimental investigations and advancements in therapeutics targeting age-related CVDs.

### 1.1 AMPK basic mechanism and function

**Figure 1:**
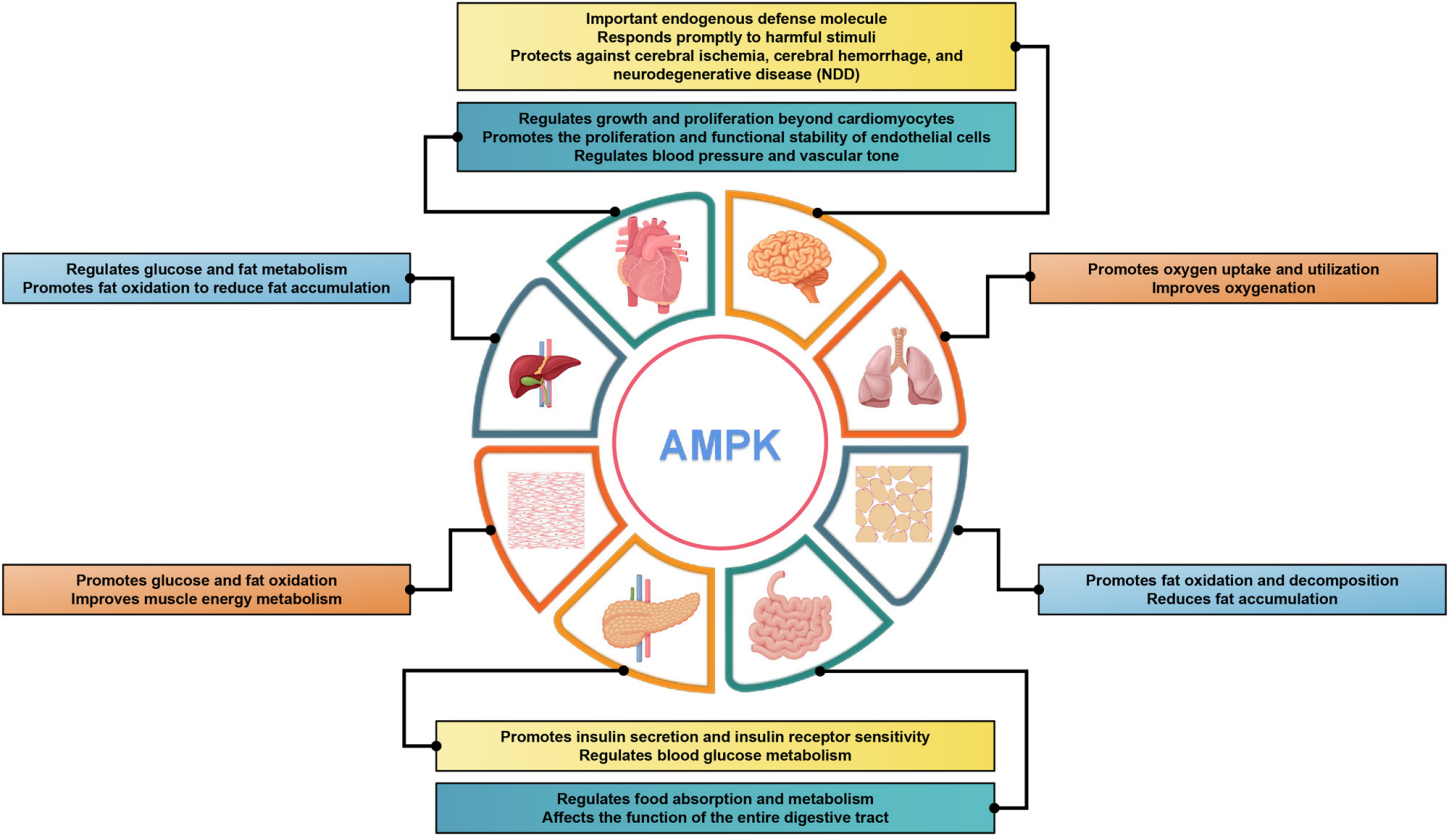
AMPK regulation in different tissues and organs. Diagram of a human body with different organs highlighted and labeled with the specific regulatory roles of AMPK in each organ. Overall, this comprehensive depiction of AMPK’s distinctive regulatory roles across various tissues and organs emphasizes its paramount importance in maintaining optimal physiological function and overall well-being. Understanding the multifaceted functions of AMPK holds potential for targeted therapeutic approaches in metabolic disorders, CVDs, neurological conditions, and respiratory ailments.

AMPK can regulates cellular energy status by promoting the adenosine triphosphate (ATP) production pathway and inhibiting the ATP utilization pathway when the body state is altered or stimulated by external factors [20]. As a highly conserved master regulator of metabolism, AMPK maintains energy homeostasis at both cellular and physiological levels during metabolic stress [21]. AMPK is commonly recognized as a precise energy sensor due to its crucial role in regulating the pathways of energy production and consumption in organisms, ensuring a dynamic equilibrium between them [22,23]. Under conditions of oxidative stress and DNA damage, AMPK regulates various cellular processes, including the inhibition of protein synthesis and cell proliferation, promotion of autophagy and DNA repair [24,25]. AMPK is a member of the serine/threonine (Ser/Thr) kinase group and is widely distributed in various cells [26,27]. It plays diverse regulatory roles in different tissues and organs. As a heterologous structured protein kinase, AMPK exerts protective effects on the heart by regulating energy homeostasis. In the brain, AMPK functions as an important endogenous defense molecule that responds promptly to harmful stimuli, such as cerebral ischemia, cerebral hemorrhage, and neurodegenerative diseases [28–30]. In the liver, it regulates glucose and fat metabolism and promotes fat oxidation to reduce fat accumulation [31,32]. In muscles, AMPK promotes glucose and fat oxidation, thereby improving muscle energy metabolism [33,34]. In adipose tissue, it promotes fat oxidation and decomposition, thereby reducing fat accumulation [35,36]. In pancreatic islets, it promotes insulin secretion and insulin receptor sensitivity and regulates blood glucose metabolism [37,38]. In the intestine, it regulates food absorption and metabolism, thereby affecting digestive tract function [39,40]. In the lungs, it promotes oxygen uptake and utilization, and improves oxygenation [41–43]. In summary, the distinctive regulatory roles of AMPK in diverse tissues and organs are crucial for maintaining normal physiological function and overall health (Figure 1).

**Figure 2:**
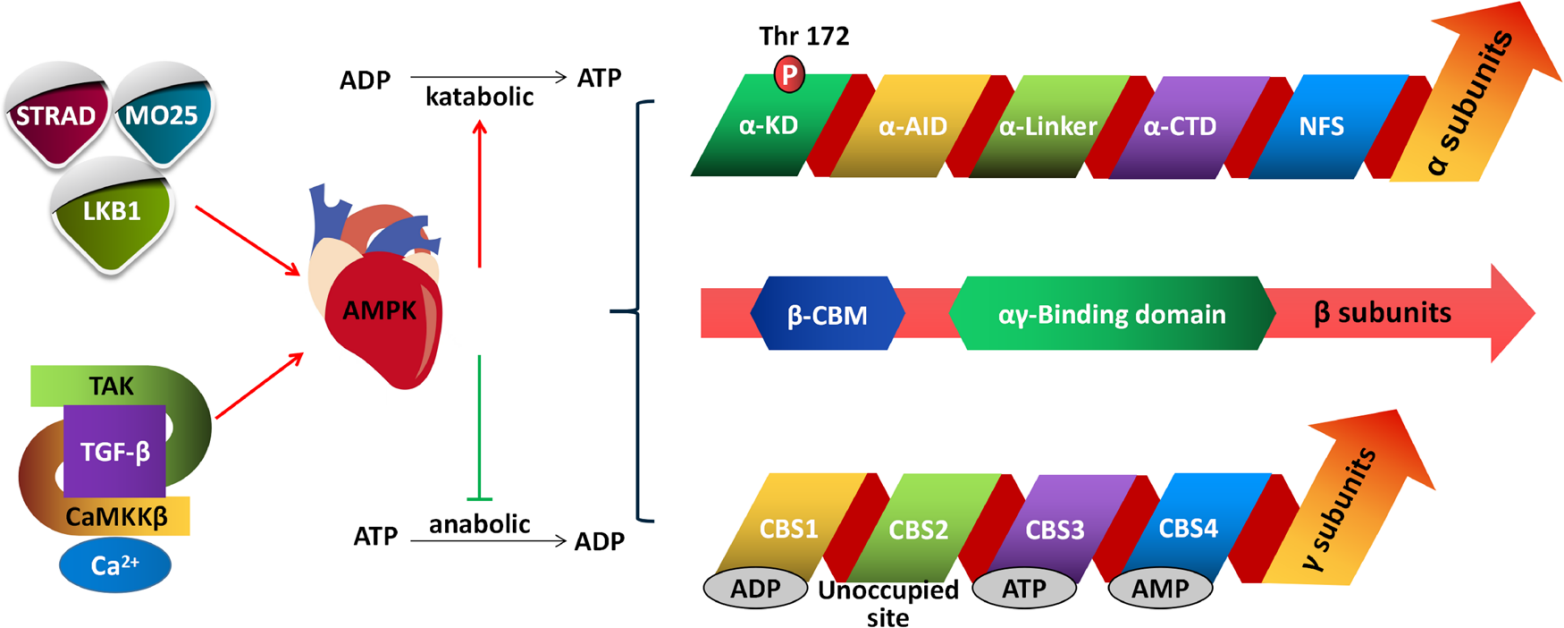
Structure of AMPK and its activation mode in the heart. There are two mechanisms through which AMPK can be activated. (1) Phosphorylation: this mechanism involves the addition of a phosphate group to the AMPK enzyme, which activates it. AMPK can be phosphorylated by several upstream kinases, including LKB1 and CaMKKβ. Once phosphorylated, AMPK becomes fully active and can regulate various cellular processes. (2) AMP/ADP binding: another mechanism of AMPK activation is through the binding of AMP or ADP molecules. When cellular energy levels drop, AMP accumulates, leading to increased binding with AMPK. This allosteric binding causes conformational changes in the enzyme, resulting in its activation. Both phosphorylation and nucleotide binding work together to activate AMPK, allowing it to modulate cellular metabolism and maintain energy homeostasis.

The varying roles of AMPK in different tissues can be primarily attributed to its complex structural composition, which consists of different subunits forming distinct structures. AMPK complexes in the body consist of three subunits: α, β, and γ. Each subunit has multiple isoforms, including α1, α2, β1, β2, γ1, γ2, and γ3, which are encoded by different genes (α1 and α2 are encoded by PRKAA1 and RKAA2 genes, β1 and β2 by PRKAB1 and PRKAB2 genes, and the three γ subunits, γ1, γ2, and γ3, are encoded by PRKAG1, PRKAG2, and PRKAB3 genes, respectively) [44]. The α subunit serves as the catalytic subunit and possesses a protein kinase structural domain. When there is an elevation in free adenosine monophosphate (AMP) and adenosine diphosphate (ADP) levels within an organism, these molecules bind to the γ subunit, inducing conformational changes in the AMPK complex. This, in turn, facilitates the phosphorylation of the α subunit threonine residue site 172 (Thr172), which represents a crucial activation pathway of AMPK [45,46]. The extent of phosphorylation at conserved threonine residues (Thr172) significantly affects the AMPK kinase activity [47]. The β-subunit is a scaffolding subunit necessary for the formation of the AMPK heterotrimer complexes. In addition, the β subunit of AMPK possesses a glycogen-binding domain, which plays a role in regulating AMPK activity thereby impacting glycogen levels within the body. β subunits are closely associated with adipogenesis, potentially due to the interaction between the β subunit of AMPK and the cystathionine-beta-synthase 2 (CBS2) structural domain of the AMPKγ subunit [48]. Interestingly, the AMPKβ subunits selectively activate AMPK complexes. For instance, specific drugs like A769622 and salicylates can selectively activate AMPK complexes containing the β1 subunit, while SUMOlation (SUMO) influences only AMPK complexes containing β2 subunit. This selective activation paves the way for the potential design of specific drugs [49–52]. These features make the AMPKβ subunit a critical factor in regulating the activity of the AMPK complex. The AMPKγ subunit has four CBS domains that are important for binding to AMP or ADP [20]. When the body has excess energy, higher levels of ATP in the body will displace AMP and bind to the AMPKγ subunit at CBS3, weakening the conformational activity of the catalytic structural domain and allowing upstream kinases and protein phosphatases to easily approach it for phosphorylation or dephosphorylation reactions. In conclusion, the AMPK phosphorylation level is an important indicator of AMPK activity [53]. Figure 2 provides a general description of AMPK structure and activity.

### 1.2 Activation of AMPK in the heart

AMPK activation is a complex process that is primarily achieved through two complementary mechanisms. First, AMPKα subunits are activated after the phosphorylation of Thr172 residues in the kinase structural domain via specific upstream kinases (liver kinase B1 [LKB1] and calcium/calmodulin-dependent protein kinase kinase beta [CaMKKβ]). However, the LKB1 pathway appears to selectively activate the complex containing the AMPKα2 subunit (but not AMPKα1) [54,55]. Mice deficient in LKB1 in skeletal and cardiac muscles exhibited loss of activity of their AMPK complex, particularly the complex containing the AMPKα2 subunit. This observation provides compelling evidence indicating that the α2 subtype plays a crucial role in protecting the myocardium against injury during ischemia [56,57]. Related studies have also elucidated the role of other important upstream kinases CaMKKβ, where an increase in intracellular Ca^2+^ concentration causes an increase in CaMKKβ activity, ultimately leading to the activation of TGF-β via transforming growth factor kinase 1 (TAK1), which ultimately culminates in the activation of AMPK through phosphorylation of Thr172. Furthermore, AMPK can be activated by heterodimerization through the binding of AMP to the AMPKγ subunit [58,59]. However, it should be noted that in cardiac tissues, the currently known expression isoforms of AMPK are α1/2, β1/2, γ1, and γ2, whereas γ3 is not expressed in the heart [60,61]. This indicates that there are several classes of AMPK variants in the heart, despite all being characterized by the presence of α, β, and γ subunits [62,63]. It has been shown that the binding of AMP to the AMPKγ subunit promotes the phosphorylation of Thr172 residues. This conformational change also protects Thr172 from dephosphorylation by protein phosphatases, including protein phosphatase 2A (PP2A), and ensures a 10-fold increase in activity [64]. AMPK activation in the heart has multiple physiological effects, including promotion of glucose uptake and oxidation, improvement of energy metabolism efficiency, enhancement of antioxidant enzyme expression and activity, reduction of oxidative stress, protection of cardiomyocytes against apoptosis, promotion of autophagy, and reduction of inflammation [65–67]. For instance, in lipopolysaccharide (LPS)-induced adipocytes, the TBK1-AMPK signaling pathway is activated and regulates the expression of omentin, which is a protein secreted by adipocytes that has multiple biological functions. Studies have shown that omentin regulates adipocyte metabolic function, promotes glucose uptake and utilization, and lowers blood glucose levels. Additionally, omentin inhibits inflammation and oxidative stress, reduces endothelial cell damage, and protects cardiovascular health [68]. Moreover, certain exogenous drugs like empagliflozin have demonstrated the ability to activate the AMPK/glycogen synthase kinase 3 beta (GSK3β) signaling pathway in diabetic cardiomyopathy. In animal experiments, this drug has shown a protective effect against cardiac injury by preventing excessive autophagy-induced cardiomyocyte death [69]. AMPK activation also regulates cardiovascular function by modulating cardiac contractility and heart rate and promoting endothelial cell function. Beyond its impact on cardiomyocytes, AMPK plays a regulatory role in the growth and proliferation of various cell types within the heart. It stimulates angiogenesis, promotes the proliferation and functional stability of endothelial cells, and regulates blood pressure and vascular tone. These functions are important for maintaining the normal physiological state of the heart [70–72]. These findings suggest that AMPK activation plays a crucial role in maintaining cardiac health and preventing CVD.

### 1.3 AMPK plays a protective role in the aging heart by regulating metabolism

Aging often leads to a progressive deterioration of cardiac geometry and systolic function; however, the exact mechanisms remain elusive [73,74]. More specifically, these changes in the heart involve a progressive decline in cardiac function and an inadequate cardiac reserve [75]. They are accompanied by myocardial hypertrophy and interstitial fibrosis, which compromise cardiac health during ventricular remodeling [6]. To date, many hypotheses have been proposed regarding the pathogenesis of cardiac aging, including inflammation, lipotoxicity, oxidative stress, apoptosis, mitochondrial damage, autophagy dysregulation, and intracellular Ca^2+^ disorders [8,76–81]. Nevertheless, given the complexity of cardiac tissues and the specific non-renewable nature of cardiomyocytes, the precise mechanisms and targets of intervention in the cardiac aging process are still in the exploratory stage [82,83]. Two signaling molecules that have been shown to be strongly regulated in age-related heart disease are protein kinase b (AKT) and AMPK, which are involved in energy metabolism [84–86]. Previously, in studies targeting obese fatty liver, it was demonstrated that AKT2 and AMPKα2, as isoforms of AKT, have an influence on obesity and hepatic steatosis induced by a high-fat diet. This suggests that Akt2 and AMPKα2 subunits have synergistic effects and may be promising therapeutic targets [87]. Contrastingly, this synergistic effect was mainly manifested by hyperactivated AKT, which promoted AMPK-Ser485 site phosphorylation and interfered with AMPK (Thr172) phosphorylation [88,89]. The synergistic effect of AKT2 and AMPKα2 subunits was also observed in the heart [90]. In particular, middle-aged mice (12 months old) with double knockout of AKT2 and AMPK showed significant changes in heart size, cardiomyocyte cross-sectional area, and interstitial fibrosis. Interestingly, this knockout did not affect Kaplan–Meier survival or the expression of senescence markers such as p16 and p21. This suggests that AKT2–AMPK knockdown does not significantly alter the biological course of cardiac senescence, but leads to a senescence-like phenotype. However, the age-related changes in myocardial contractile function caused by AKT2–AMPK knockout can be attributed to mitochondrial dysfunction. These changes can be indicated by alterations in mitochondrial structural genes (UCP2, PGC-1α, and electron microscopic ultrastructure), autophagic genes (Beclin-1, LC3B, Atg5, Atg7, and p62), phagocytic genes (PINK1, Parkin, Fundc1, and BNIP3), and lysosomal biogenesis related gene (TFEB). These findings suggest a critical role for AMPK–AKT-mediated autophagy in altered cardiac geometry and function [91,92]. Furthermore, AKT2–AMPK knockdown fails to alter the intracellular processing of Ca^2+^ levels at a young age, which eventually manifests as defective intracellular Ca^2+^ processing in cardiac myocytes with the progression in age leading to myocardial contractile dysfunction [93]. In addition, the downregulation of autophagy mediated by the senescence-associated AMPK-S-phase kinase associated protein 2 (SKP2)-coactivator associated arginine methyltransferase 1 (CARM1) pathway also leads to cardiomyocyte dysfunction [94,95]. Much has been reported regarding the role of autophagy in the regulation of cardiac function; however, there are dramatic differences between young and old hearts. For instance, light fasting affects the autophagic flux differently in young and old hearts [96,97]. CARM1 stability is significantly reduced in the aging heart, leading to impairment of the nuclear TFEB–CARM1 complex and autophagic flux [92]. With a reduction in AMPK–FOXO3 activity in the nucleus, it is unable to inhibit SKP2-E3 ubiquitin ligase. However, this failure of inhibition can be restored by the activation of AMPK. Nevertheless, excessive activation of the AMPK–SKP2–CARM1 pathway may also lead to cardiomyocyte hypertrophy [98]. As a macroscopic means of regulating metabolism, calorie restriction can extend the average and maximum life span and has beneficial effects on age-related diseases [99–101]. Although calorie restriction may lead to different intervention outcomes in younger hearts, calorie restriction in middle-aged or older populations has been effective in preventing CVDs associated with aging [102,103]. A study has shown that starting a calorie-restricted diet for 3 months (40% less than *ad libitum*) in 12- and 19-month-old mice significantly reversed aging-related markers, including p16 and p21, and significantly improved markers of cardiac remodeling (cardiac hypertrophy and myocardial fibrosis), inflammation, mitochondrial damage, and telomere shortening [104]. The analysis of related miRNAs and corresponding target genes revealed that this result was likely due to a significant increase in the phosphorylation level of AMPK (Thr172) in the heart under caloric restriction, which regulates the expression of FOXO transcription factors and, subsequently, the expression of autophagy-related genes to achieve cardioprotection [105,106]. Autophagy plays an important role in the pathogenesis of atherosclerosis and other age-related diseases. In the heart, C1q/TNF-related protein 9 (CTRP9) has anti-aging and anti-atherosclerotic effects that highly resemble lipocalin in structure, while AMPK plays a positive role in cardioprotection mediated by CTRP9 [107,108]. Activated AMPK is involved in LC3 conversion and reduces the levels of p62 induced by CTRP9. Conversely, CTRP9 restores autophagy and autophagic flux via AMPK activation, thereby inhibiting endothelial senescence produced by palmitic acid [109]. Similarly, the role of AMPK in LPS-induced myocardial dysfunction is similarly age-related [110]. The AMPK/mTOR (mammalian target of rapamycin, mTOR) pathway serves as a potential mechanism for regulating autophagic function in mice of different ages, and its impairment or deficiency leads to significant alterations in the mouse heart, including echocardiography, pathology, contractility, and intracellular Ca^2+^ levels [111]. However, A769662, an AMPK agonist, appeared to have a better regulatory function in aged mice [112]. When the expression of the AMPK upstream regulators PP2A and PP2Cα was significantly increased, AMPK activity was inhibited, mTOR was activated, and autophagy was inhibited. In contrast, the addition of the AMPK activator A769662 significantly decreased the expression of p-mTOR and p-S6, increased the levels of autophagy markers Atg5 and p62, and the LC3-II/LC3-I ratio, ultimately improving cardiac function and upregulating cardiac autophagy levels under LPS in aged mice [113]. In addition, it has been shown that increased CD36 in aged male mice may reduce AMPK activity, leading to activation of the mTOR-p70S6K pathway and causing myocardial hypertrophy [114].

### 1.4 AMPK and sirtuin family co-regulate cardiac function and aging

A common feature of heart disease and aging is the alterations in metabolic organs, which ultimately lead to changes in circulating metabolite levels [115]. AMPK, an important regulator of energy homeostasis, emerges as a crucial player in aging-induced metabolic dysregulation. For instance, a study has shown that NADPH pretreatment of neonatal rat cardiomyocytes significantly increased AMPK phosphorylation while downregulating mTOR phosphorylation and effectively inhibiting hypoglycemic hypoxia/reoxygenation OGD/R(oxygen-glucose deprivation/reoxygenation)-induced apoptosis [116]. Conversely, NADPH-induced AMPK phosphorylation and cardioprotection were blocked when AMPK, which inhibits mitochondrial damage and cardiomyocyte apoptosis, was inhibited by compound C (Dorsomorphin) [117].

The Nmrk2 gene is a co-responsive gene for AMPK and PPARA; additionally, in isolated rat cardiomyocytes, energy stress and high NAD+ depletion will activate the Nmrk2 gene [118]. Moreover, promoting the synthesis of NAD+ can effectively stimulate glycolysis in cardiomyocytes, increase AMPK activity, and improve or reduce the development of heart failure in mice [119]. Of note, NAD is involved in cellular metabolism and DNA repair through its role as a sensing or consumable molecule for the enzymes poly (ADP-ribose) polymerase 1 (PARP1) and sirtuin protein deacetylases, and these deacetylations directly or indirectly regulate cellular aging and inflammatory responses [120,121].

Aging, as an important risk factor for left ventricular hypertrophy and CVD development, can be effectively mitigated by targeting AMPK. The sirtuin family of nicotinamide adenine dinucleotide-dependent deacetylases (SIRT1-7) plays a significant role in improving cardiac metabolism and maintaining essential cardiac functions [122]. Most sirtuin family proteins delay aging to some extent when their expression is upregulated in the heart. Age-related changes in SIRT1, AMPK, and SIRT3 are associated with mitochondrial biogenesis, antioxidant defense, and cardiac inflammation. Among them, AMPK and SIRT1 are partner proteins that coordinate multiple intracellular processes, including cellular resistance to oxidative stress, general metabolism, inflammation, and mitochondrial biogenesis and function, while overexpression of SIRT3 activates the AMPK pathway and improves mitochondrial biogenesis, which is required to maintain mitochondrial redox homeostasis, sustain mitochondrial respiration, and inhibit mitochondrial apoptosis [123,124]. SIRT2 promotes downstream AMPK activation by deacetylating LKB1, a kinase upstream of AMPK [125]. However, the physiological and pathological roles of SIRT4 in cardiac aging are unknown. In response to DOX-induced cardiotoxicity, SIRT3 and SIRT4 increase autophagy through the AMPK/mTOR signaling pathway, while activation of the FOXO and P53 pathways to reduce apoptosis may be a joint action of SIRT3 and SIRT4 as well as AMPK [126]. The absence or abnormality of SIRT5, a key enzyme regulating mitochondrial function in the heart, leads to the inhibition of mitochondrial NADH oxidation and ATP synthase activity. Interestingly, when SIRT5 is blocked, with reduced ATP levels and an increased AMP/ATP ratio, AMPK is activated to a great extent. In a SIRT5 knockout mouse model, this was accompanied by elevated phosphorylation AMPK (Thr172), which alleviates left atrial dilation, a structural change in the aging heart [127,128]. Moreover, unlike the conventional perception of reduced expression of plasminogen activator inhibitor-1 (PAI-1) mediated by Jun N-terminal kinase (JNK) and p38, the inhibition or silencing of SIRT5 inhibits the expression of PAI-1 genes and proteins during thrombosis by increasing AMPK activation and reducing the phosphorylation of mitogen-activated protein kinase and extracellular signal-regulated kinase 1/2 (ERK 1/2 kinase), ultimately achieving a response to TNF-α and reducing thromboembolic episodes [129]. SIRT6, a crucial member of the sirtuin family, plays an essential role in regulating DNA repair, telomere maintenance, and glucose and lipid metabolism [130]. In the heart, the melatonin membrane receptor-mediated SIRT6–AMPK–PGC-1α–AKT axis may be a potentially effective strategy to attenuate dilated cardiomyopathy and reduce the myocardial response to ischemia/reperfusion injury (I/RI) in patients with diabetes [131]. With the activation of SIRT6, it significantly increases nocturnal circulating melatonin and cardiac melatonin levels, while elevated melatonin levels in the heart activate the downstream AMPK–PGC-1α–AKT axis, ultimately improving the outcome of I/RI [132]. In *in vitro* experiments, sustained activation of AMPK increased the mRNA and protein expression of Troponin T type 2 (TNNT2) and Troponin I type 3 (TNNI3), maintained the stability of myocardial muscle contraction, and enhanced the activity of SIRT1 and SIRT6 by decreasing histone acetylation [133]. SIRT7 is a histone H3K18-specific deacetylase that epigenetically controls mitochondrial biogenesis, ribosomal biosynthesis, and DNA repair [134]. There is a relative lack of research on the role of SIRT7 in cardiac aging, and previous studies that have been conducted have presented different conclusions. A study has shown that SIRT7 deficiency protects against aging-associated glucose intolerance and extends lifespan in male mice [135]. However, other studies have shown that SIRT7-deficient mice exhibit several signs of aging, including degenerative cardiac hypertrophy, kyphosis, reduced subcutaneous fat, and poor stress resistance [136,137]. Interestingly, proteasome activator subunit 3 (REGγ) has been reported to promote SIRT7 degradation in an AMPK phosphorylation-dependent manner [138]. However, fasting coordinates AMPK and GSK3β activity to ensure the stabilization of SIRT7. More precisely, AMPK phosphorylates SIRT7 at T263 to prime subsequent phosphorylation at T255/S259 by GSK3β, decoupling SIRT7 from UBR5 E3 ligase, and thereby preventing K48-linked polyubiquitination and proteasomal degradation of SIRT7 [139].

### 1.5 AMPK inhibits SASP transmission between cells and resists damage to the heart from excessive reactive oxygen species (ROS)

**Figure 3:**
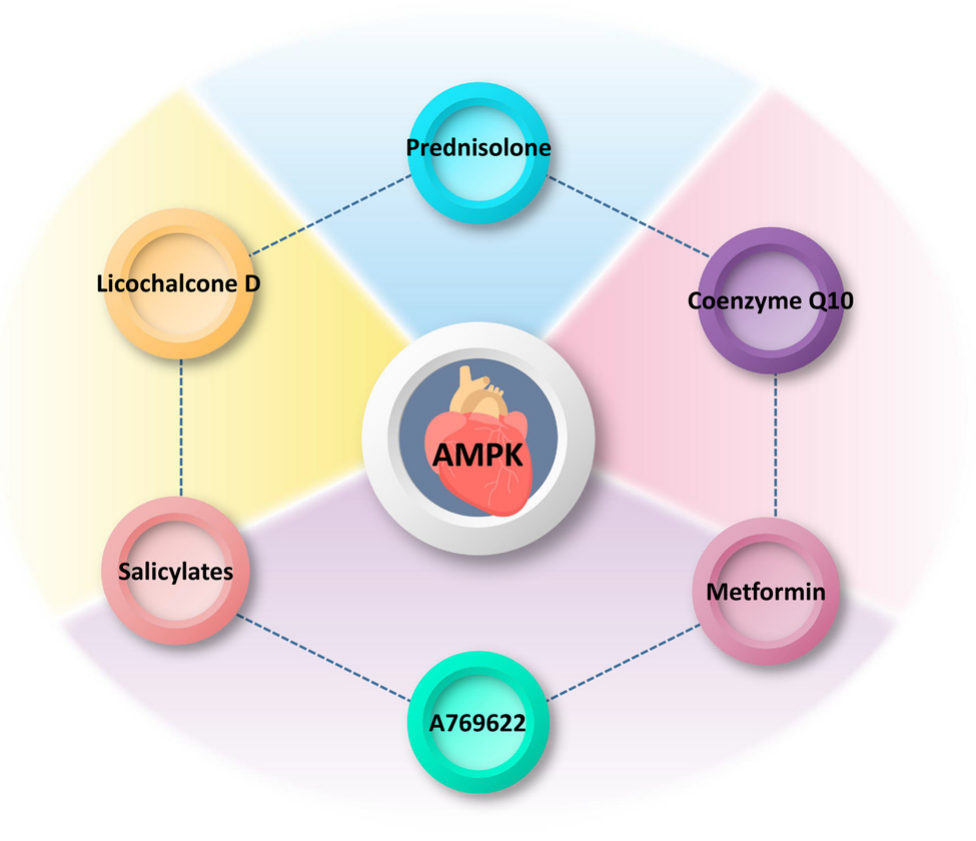
Interactions of molecules/drugs with AMPK in the heart. The diagram illustrates several molecules/drugs that interact with AMPK specifically in the heart, a crucial organ involved in cardiovascular health. Prednisolone: a synthetic corticosteroid commonly used as an anti-inflammatory and immunosuppressant agent. Prednisolone may modulate the activity of AMPK in the heart, potentially influencing downstream signaling pathways involved in cardiac metabolism, inflammation, and oxidative stress. Licochalcone D: a natural flavonoid found in licorice root, known for its antioxidant and anti-inflammatory properties. Licochalcone D has been reported to activate AMPK in the heart, suggesting its potential cardioprotective effects by enhancing cellular energy regulation, reducing oxidative damage, and modulating inflammatory responses. Salicylates: salicylates have been shown to activate AMPK in the heart, indicating their potential in ameliorating cardiac dysfunction through effects on glucose metabolism, mitochondrial function, and oxidative stress. Coenzyme Q10: a naturally occurring compound involved in cellular energy production. Coenzyme Q10 has been reported to regulate AMPK activity in the heart, potentially improving myocardial energy metabolism, protecting against oxidative damage, and promoting overall cardiac health. Metformin: a widely prescribed drug for the treatment of type 2 diabetes. Metformin activates AMPK in the heart, leading to beneficial effects on glucose metabolism, mitochondrial function, calcium handling, and overall cardiac performance. A769622: an experimental small molecule compound targeting AMPK activation. A769622 has been studied for its potential cardioprotective effects by enhancing cardiac energy metabolism, attenuating hypertrophy, improving contractility, and reducing cardiac ischemic injury.

Cellular senescence is closely related to the SASP of inflammatory and secretory proteins. The close transfer of SASP between cells is a major trigger for cellular senescence, in other words, SASP secretion inevitably increases with age, and inhibiting or eliminating SASP secretion and activity is one of the important ways to combat aging [140,141]. Cardiovascular smooth muscle cells (VSMCs) in the heart are the main cells that express SASP, which promotes chronic vascular inflammation, loss of vascular function, and the development of age-related heart diseases. Prednisolone inhibits p-NF-κB via the SIRT1 and p-AMPK (Ser485) pathways, ultimately resisting VSMC aging and inflammatory responses [142]. Another drug, metformin, has a potential resistance to aging-related injuries and can improve mitochondrial function to mitigate ischemia–reperfusion damage to the heart and effectively resist myocardial necrosis [143]. As an known AMPK receptor agonist, it inhibits LPS-induced chemokine expression via the AMPK and NF-κB signaling pathways, including CCL2, CXCL10, and CXCL11, which are all chemokines in the SASP [144,145]. Recent *in vitro* experiments have revealed that Licochalcone D (Lico-D)-mediated autophagy activation through the upregulation of AMPK may reduce H_2_O_2_-induced oxidative stress-induced senescence [24]. Figure 3 shows several molecules/drugs that interact with AMPK. *In vivo* experiments showed that the antioxidant, anti-aging, and cardioprotective effects of Lico-D may arise through the activation of AMPK and autophagy and ameliorate oxidative stress-induced aging. Along with AMPK activation, the expression levels of senescence markers (such as p53 and p21) were also significantly downregulated [146]. Excess mitochondrial production of ROS free radicals is directly and causally linked to aging of the heart and other organs and plays a deleterious role in several types of age-related cardiac diseases, including I/RI and heart failure, which occur in a high proportion of elderly patients [147]. With the gradual refinement of the ROS theory of oxidative stress, ROS production is also considered a fundamental mitochondrial function that coordinates several signaling pathways to exert beneficial effects, some of which are protective in the heart [148,149]. However, aging cardiomyocytes undergo cytoarchitectural and physiological changes as a result of their timely response to exercise, stress, injury, and their own reduced adaptive reserve capacity. At this time, the disturbed redox state may synergistically promote the production of mitochondrial ROS and exacerbate cardiomyocyte death in the elderly heart. As the heart ages, leading to elevated levels of ROS in the body, a feedback mechanism is triggered, and the expression of antioxidant enzymes is stimulated, which is inherent to the heart. The main player in this process is the transcriptional coactivator, PPARγ coactivator 1α (PGC-1α), which is a regulator of mitochondrial biogenesis and one of the important inducers of antioxidant gene expression during oxidative stress; however, PGC-1α levels are low in aging cardiac myocytes. AMPK can directly phosphorylate PGC-1α in skeletal muscle and, using positive feedback triggering its own transcriptional activation. It can be argued that when normal homeostatic metabolic mechanisms are disrupted, AMPK is activated and stimulates the expression of antioxidant enzymes to limit the production of ROS [150]. Previous reports have proposed that AMPK activity is affected by ROS, mainly through the action of ROS on redox-sensitive cysteine residues (Cys-299/Cys-304) on the AMPKα subunit, which increases AMPK activity [151]. However, it has also been suggested that altered AMPK activity in response to redox changes is not due to the regulation of AMPK *per se*, but is a secondary result of redox effects on other processes, such as mitochondrial ATP production [152]. In cardiomyocytes, most ROS are generated by electron leakage from the mitochondrial electron transfer chain (ETC) [153]. Age-related changes in mitochondrial function and decreased ETC complex activity lead to higher ROS production rates associated with oxidative stress, ultimately leading to cardiac aging. NOX, an NADPH oxidase, is a major contributor to ROS production in the cardiovascular system [154]. The reason for the production of ROS by nicotinamide adenine dinucleotide phosphate oxidase (NOX) lies in its specific function in the transmembrane electron transport of superoxide anions produced by immune cells [155]. All seven isoforms of NOX are expressed in the vascular smooth muscle, with Nox1, Nox2, Nox4, and Nox5 being the most abundant in relative terms [156]. NOX plays a crucial role in physiological and pathological processes, as it physiologically produces ROS necessary to maintain cardiovascular homeostasis; however, the abnormal production of ROS often originates from this and is accompanied by the onset of accelerated aging. In fact, there is considerable controversy regarding the harmful or non-harmful functions of NOX. When AMPK knockdown increases Nox2 protein expression, and conversely, AMPK agonists decrease Nox2 expression, AMPK can directly or indirectly resist Nox2-associated oxidative stress, leading to I/RI exacerbation [117,157]. In addition, in a pressure overload model, Nox2 activation led to cardiac systolic dysfunction and interstitial fibrosis formation [158]. However, elevated endothelial Nox4-derived ROS promote endothelial cell migration and angiogenesis in an endothelial nitric oxide synthase (eNOS)-dependent manner during ischemia and can protect the heart to some extent [159]. Additionally, endogenous coenzyme Q10 can improve the functionality of endothelial precursor cells by increasing eNOS activity and nitric oxide production. Through CAMKK activation of AMPK, it promotes the expression of eNOS and Heme Oxygenase-1, thus improving cellular apoptosis induced by high glucose and mitochondrial membrane potential imbalance [160,161]. Interestingly, there is also evidence that metformin activates AMPK and inhibits NOX4 expression, leading to reduced myocardial oxidative damage and apoptosis, thereby attenuating reperfusion injury [162,163]. Overall, although the effects of NOX and its isoforms and their corresponding derivatives on the heart are still controversial, the effects of AMPK against NOX are mostly favorable for survival.

## 2 Conclusions

**Figure 4:**
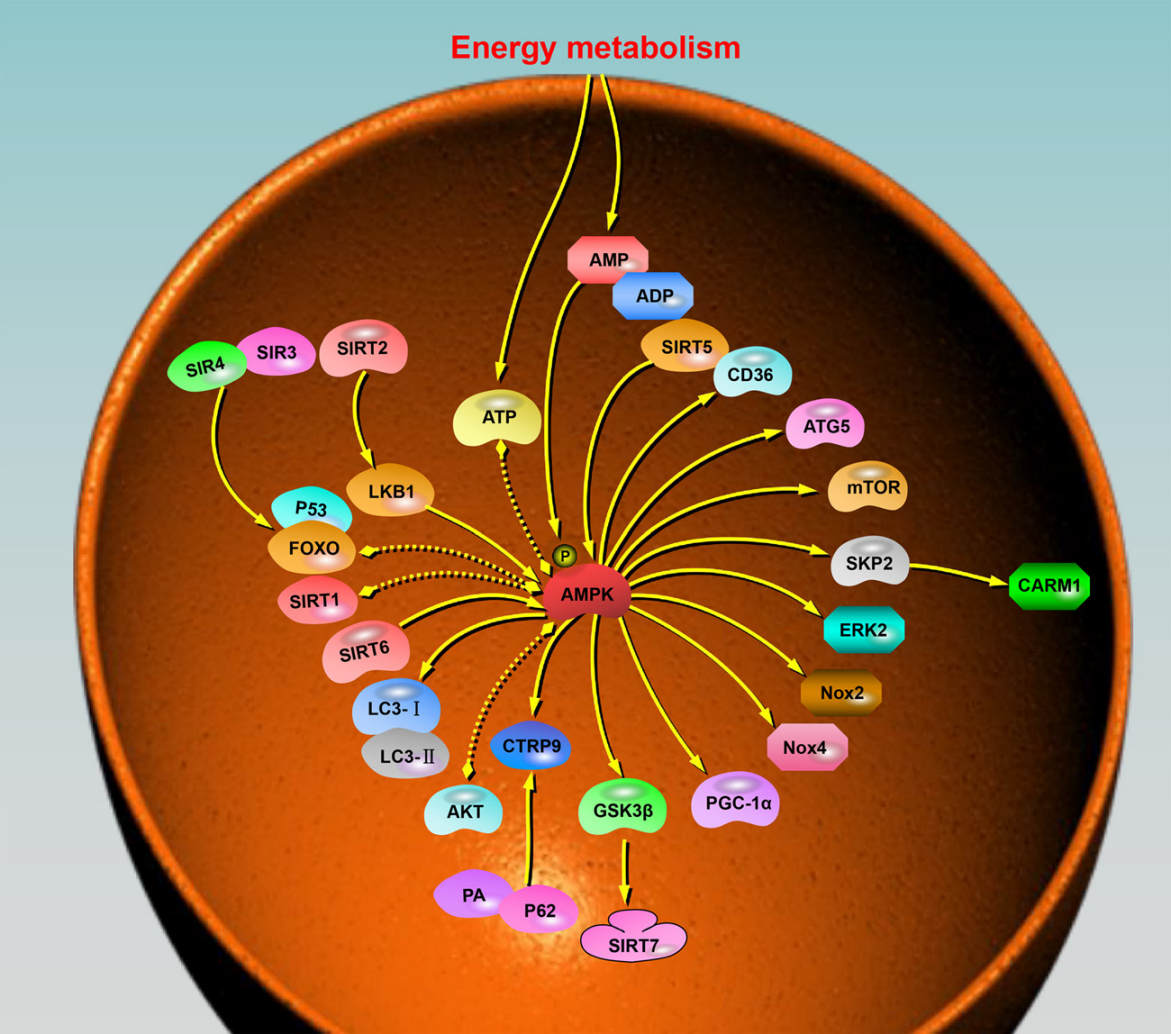
Molecular pathways triggered by or associated with AMPK during cardiac aging. AMPK is crucial in regulating energy metabolism, autophagy, and protein synthesis in the heart. The diagram illustrates the interactions of multiple molecules with AMPK. The arrows within the diagram indicate the general direction of action or influence between AMPK and the respective molecules. These arrows represent the regulatory effects exerted by AMPK on the molecules or vice versa. Furthermore, the presence of dotted double arrows in the diagram signifies the existence of mutual regulation between AMPK and the associated molecules. This mutual regulation suggests a bidirectional influence, where AMPK affects the activity or function of the molecules, while the molecules, in turn, impact AMPK signaling or activity.

Globally, it is estimated that the number of individuals with CVD will reach 1.9 billion, and there is a significant correlation between age and morbidity/mortality. Therefore, it is crucial to understand the molecular mechanisms of cardiac aging and the important pathways that can influence cardiac function during aging to develop interventions that target these mechanisms. AMPK is required for embryonic development, growth, and maintenance of the physiological functions of several organs, including the heart. The pathophysiological functions of the AMPK pathway as a central energy regulator in aging, particularly cardiac aging, have been extensively studied. When activated in response to nutritional signals, AMPK increases the body’s energy reserves, promotes cell growth, and regulates autophagy to a certain extent. Conversely, AMPK which is overexpressed or affected by inhibitors, inhibits the overall protein translation and reduces autophagic flux, ultimately affecting protein quality. The role of AMPK in the heart varies across different life stages. In the aged heart, AMPK exhibits the ability to effectively block the *in vivo* transmission of SASP signals emanating from senescent cells. Moreover, AMPK activates autoimmune cells to eliminate senescent cells, thus preventing contact-induced aging. These beneficial effects involve mechanisms like autophagy. Furthermore, the reciprocal regulation with the sirtuin family and enhanced resistance to ROS are likely to promote an overall extension of lifespan. Although the regulation of AMPK is stable *in vivo* and is hardly overexpressed, studies on AMPK isoforms in different tissue sites of the heart need to be improved, and the advantages and disadvantages of various modes of AMPK activation also need to be investigated. The molecular pathways initiated or influenced by AMPK and associated with cardiac aging are summarized in Figure 4. In conclusion, AMPK holds significant potential as a therapeutic target for addressing age-related cardiac diseases and combating the aging process. However, further research is warranted to investigate ways to activate AMPK, specifically in cardiac tissues, and to investigate the development of specific agonists for this purpose.
